# A distinct species, *Dodona formosana*, detected in the *Dodona eugenes* species complex: clarification of the taxonomic status of the Punch butterfly in Taiwan

**DOI:** 10.3897/zookeys.736.22062

**Published:** 2018-02-08

**Authors:** Li-Wei Wu, Wen-Jie Lin, Yu-Feng Hsu

**Affiliations:** 1 The Experimental Forest, College of Bio-Resources and Agriculture, National Taiwan University, Nantou, Taiwan; 2 Department of Life Science, National Taiwan Normal University, Taipei, Taiwan

**Keywords:** endemism, male genitalia, Myrsinaceae, wing pattern

## Abstract

The Tailed Punch, *Dodona eugenes*, is widely distributed in East Asia with seven subspecies currently recognized. However, two of them, namely ssp. formosana and ssp. esakii found in Taiwan, are hard to distinguish from each other due to ambiguous diagnostic characters. In this study, their taxonomic status is clarified by comparing genitalia characters and phylogenetic relationships based on mitochondrial sequences, COI and COII (total 2211 bps). Our results show that there is no reliable feature to separate these two subspecies. Surprisingly we found that *Dodona* in Taiwan is more closely related to the Orange Punch, *D. egeon*, than to other subspecies of *D. eugenes*. Therefore, the following nomenclatural changes are proposed: *Dodona eugenes formosana* is revised to specific status as *Dodona formosana* Matsumura, 1919, **stat. rev**, and ssp. esakii is sunk to a junior synonym of *Dodona formosana* **syn. n.**

## Introduction

*Dodona eugenes* Bates 1868 is a medium-sized metalmark butterfly, distributed in East Asia from Muri (Pakistan), Nepal, north India, Bhutan, Indochina, western China, Hainan to Taiwan. Seven subspecies were recognized (Fruhstorfer 1912; Matsumura 1919; Shirôzu 1952; D’Abrera 1986; Gu and Chen 1997; Chou and Yuan 2000), with two of them endemic to Taiwan, viz. ssp. formosana Matsumura 1919 and ssp. esakii Shirôzu 1952 (Shirôzu 1960; Igarashi and Fukuda 2000; Hsu 2013). Shirôzu (1952) noted that the body size and the wing pattern are different between northern and central populations in Taiwan, and described the small body size population in central Taiwan as ssp. esakii. The larval hostplants were subsequently documented as Myrsine africana (Myrsinaceae) for ssp. esakii (Lin 2004) and M. seguinii for ssp. formosana (Hsu 2006).

Shirôzu (1952) described ssp. esakii, but also mentioned that its diagnostic features are not always present. Moreover, when more *Dodona* populations were later discovered from southern and eastern part of Taiwan, all of them were arbitrarily assigned to ssp. esakii (Hamano 1987; Lee and Wang 2007; Hsu 2013; Lin and Su 2013; Lin 2016), even though some individuals from these regions show characters similar to ssp. formosana (Lin 2016; Chen 2017; NTNU specimens), blurring the distinction of the two subspecies. To this date, no effort has been made to compare the genitalia within these two groups or among other subspecies of *Dodona eugenes*, although morphology of genitalia is usually considered the most important character set for species identification in Lepidoptera (Scoble 1992).

The distribution of the two putative *Dodona* subspecies in Taiwan is still difficult to document based on literature (Yamanaka 1975). No clear geological boundary exists, suggesting the possibility that the differentiation between the two putative subspecies may be caused by other factors, such as utilization of different hostplants which may facilitate diversification of herbivorous insects (Linn et al. 2003; Braby and Trueman 2006; McBride et al. 2009; Nylin and Janz 2009). However, the ranges of the two putative subspecies and their presumable hostplants do not fully match, e.g., the ssp. formosana is only distributed in northern part of Taiwan, but its known hostplant *M. seguinii* is found all the way to southern part of Taiwan (Yang and Lu 1996). Other physiological factors may be also involved, as adult size and reproductive strategies of herbivores insects may be affected by the nutrient content or quality of their hostplants (García‐Barros 2000; Awmack and Leather 2002).

The *Dodona eugenes* species complex was proposed for a few closely related species, which share similar wing markings as *D. eugenes* (Lin 2016). Currently, seven subspecies are recognized, which are widely distributed in East and Southeast Asia. However, the species-level taxonomy of *D. eugenes* has been problematic, with one of the former subspecies, spp. *maculosa*, recently recognized as a distinct species, *D. maculosa* based on morphology of male genitalia (Callaghan 2009). To verify the taxonomic status of the *Dodona* butterfly in Taiwan, the morphology of the male genitalia was examined as well as DNA-based phylogenetic relationships.

## Materials and methods

### Sampling

To verify taxonomic status of the *Dodona* butterfly in Taiwan, a total of 92 riodinid individuals was sampled for morphological and molecular analyses (Suppl. material 1), including 53 *Dodona* specimens from Taiwan (15 spp. *esakii*, and 38 spp. *formosana*), eleven *D. maculosa*, nine *D. eugenes* (eight ssp. eugenes, and one spp. *venox*), seven *D. egeon*, four *D. adonira*, two *D. ouida*, two *Takashia nana*, two *Polycaena chauchawensis*, one *P. princeps*, and one *D. elvira*. *Polycaena* and *Takashia* were used as outgroups based on the previous phylogenetic relationships of Riodinidae (Espeland et al. 2015). Vouchers are deposited in the following institutions.

Abbreviations for depositories:

**NTNU** National Taiwan Normal University, Taipei, Taiwan

**NMNS** National Museum of Natural Science, Taichung, Taiwan

**SEHU** Systematic Entomology Laboratory, Faculty of Agriculture, Hokkaido University, Japan

### Morphology of genitalia

In total, 65 riodinid specimens were examined for male genitalia morphology, including 27 spp. *formosana*, eleven spp. *esakii*, ten *D. maculosa*, five *D. eugenes eugenes*, four *D. egeon*, three *D. adonira*, one *D. eu. venox*, one *D. ouida*, one *Takashia nana*, and one *Polycaena chauchawensis* (Suppl. material 1). Abdomens were first placed in 70 % alcohol, and soft tissue was dissolved by macerating the abdomen in a 10 % NaOH aqueous solution for 5–8 minutes. The macerated abdomens were transferred to 70 % alcohol for genitalia dissection and subsequently preserved in 70 % alcohol together with chlorazol black. The phallus was separated from the other parts before being mounted on a slide in euparal. Genitalia slides were named by the genus name *Dodona* (Dn). Terminology of genitalia follows Klots (1970) and Kristensen (2003). The length of uncus, valva, and phallus were measured by Image-Pro Plus 5.1 (Media Cybernetics, Silver Spring, MD) and statistics were performed using JMP 5.0 (SAS Institute, Cary, NC).

### Molecular procedures

DNA was extracted from two legs or thorax muscle using the Puregene DNA Isolation kit (Gentra Systems, Minnesota, USA). Mitochondrial *cytochrome c oxidase 1* (COI) and *cytochrome c oxidase 2* (COII) genes were amplified using the primers listed in Supplementary file 2. Polymerase Chain Reaction (PCR) was performed in a 30 μL volume, containing 23.5 μL of sterile ddH_2_O, 1 μL of extracted DNA, 1 μL of 10 μM dNTP, 3 μL of 10X PCR reaction buffer, 0.6 μL of each 10 μM primer, and 0.3 μL of Power Taq (Genomics Biosci & Tech, Taiwan). PCR was carried out using two settings as follows: (1) Standard: initial denaturation of 5 mins at 95 °C, followed by 40 cycles consisting of denaturation of 30 s at 95 °C, annealing of 30 s at 57–47 °C, and extension of 30–60s at 72 °C, and final extension of 7 mins at 72 °C; (2) Touchdown: initial denaturation of 5 mins at 95 °C, followed by 20 cycles consisting of three steps of 30 s at 95 °C, 30 s at 65–55 °C (-0.5 °C per cycle), and 30 s at 72 °C, and then additional 20 cycles consisting of 30 s at 95 °C, 30 s at 55–45 °C, and 30 s at 72 °C, and final extension of 7 mins at 72 °C. The quality of PCR products were visually checked on 1–2% agarose gels. If DNA fragments were correctly amplified, the PCR products were sequenced using an ABI 3730 DNA Analyzer (Applied Biosystems, Foster City, CA, USA).

### General sequence information

DNA sequences were checked and corrected by eye using Sequencher 4.10 (Gene Codes, Ann Arbor, USA). Sequence matrices were aligned using MUSCLE (Edgar 2004), and the aligned datasets were saved in the fasta or nexus format for subsequent analyses. Genetic distances were calculated using Kimura-2-parameter models implied by MEGA 6.0 (Tamura et al. 2013), and general sequence information was calculated using web server DIVEIN (Deng et al. 2010). All sequences were submitted to GenBank under the accession numbers KX866690-KX866733 (listed in Suppl. material 1).

### Phylogenetic analyses

Molecular phylogenies were reconstructed under the Bayesian inference (BI) and Maximum Likelihood (ML) criteria. The BI analysis was performed in MrBayes v. 3.2.6 (Ronquist et al. 2012). The best-fit data partitioning and substitution models (Table 1) were selected using the results produced by PartitionFinder v 1.1.1 (Lanfear et al. 2012). Two independent runs for three partition schemes were performed with eight chains (seven heated and one cold), and five million generations with sampling every 100 generations were set. The first 25 % of generated trees were discarded as burn in (default setting) and the remaining trees were used for producing a majority rule tree with posterior probability for each nodal support. To check the quality of our Bayesian phylogenies, the effective sample size (ESS) of each parameter was over 200, and the convergence test of Marko Chain Monte Carlo (MCMC) chains was checked by Tracer 1.6 (Rambaut and Drummond 2013). The ML analysis was done using RAxML Pthreads-based version 8 (Stamatakis 2006; Ott et al. 2007), and the optimal substitution model and partitioning schemes were found using PartitionFinder (Table 1). ML analysis was done with three partitions under the GTRGAMMA model, with 1000 replications for calculating bootstrap support values. *Takashia nana* was used as a single outgroup in both BI and ML analyses.

**Table 1.** Partitions and substitution models used in this study. Substitution models for ML method were reduced to GTR+G.

**Method**	**Partition nos.**	**Partitions and substitution models**
Bayesian inference	3	COI position 1 + COII position 1 + COII position 2 (HKY+I), COI position 2 (F81), COI position 3 + COII position 3 (GTR+I)
Maximum Likelihood	3	COI position 1 + COII position 1 (GTR+I+G), COI position 2 + COII position 2 (GTR+G), COI position 3 + COII position 3 (GTR+I+G)

### Haplotype network

To examine the genetic structure of *Dodona* in Taiwan, a haplotype network was constructed using the TCS 1.21 software (Clement et al. 2000), based on maximum parsimony (Templeton et al. 1992).

## Results

### Morphology of genitalia

**Figure 1.**
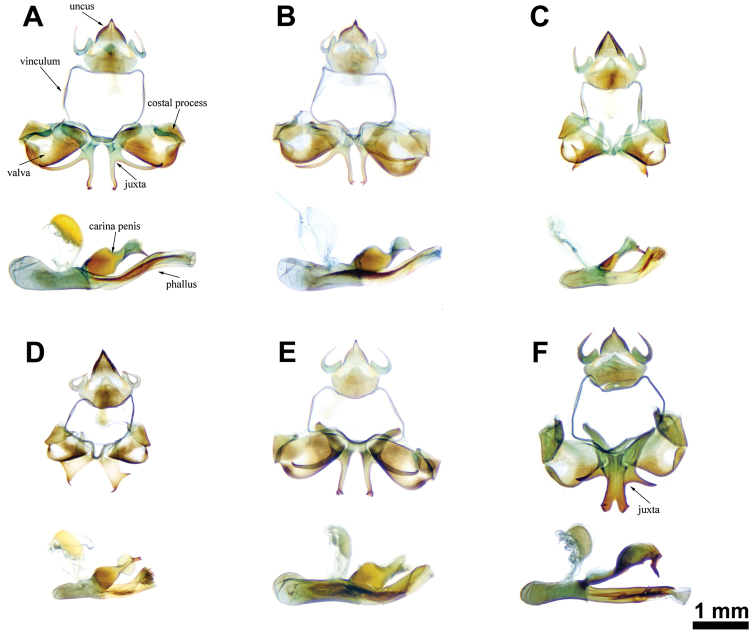
Male genitalia of *Dodona* samples. **A** *D. formosana* (spp. *formosana*) **B** *D. formosana* (spp. *esakii*) **C** *D. eugenes eugenes* **D** *D. eugenes venox* **E** *D. egeon* **F** *D. maculosa*.

**Figure 2.**
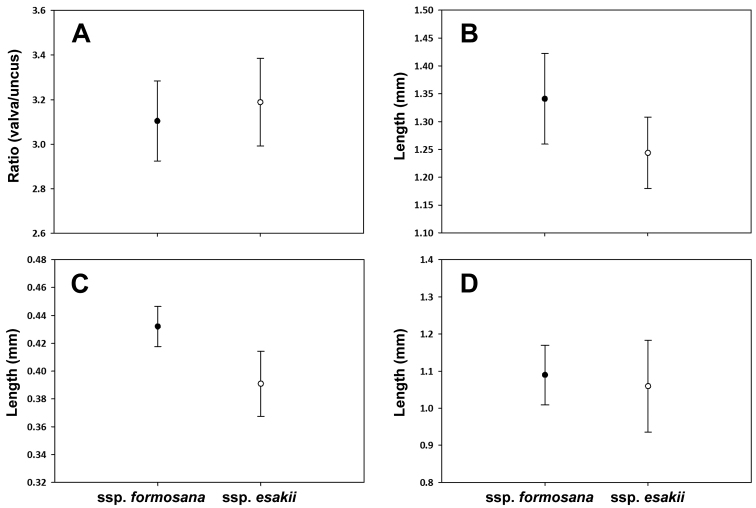
Morphological comparison of genitalia between ssp. formosana and ssp. esakii. **A** Shape of genitalia (t-test, t = -0.9868, d.f. = 18, p = 0.3368) **B** Valva (t-test, t = 2.9904, d.f. = 18, p < 0.05) **C** Uncus (t-test, t = 4.6152, d.f. = 18, p<0.001) **D** Phallus (t-test, t = 0.6356, d.f. = 18, p = 0.5331).

**Figure 3.**
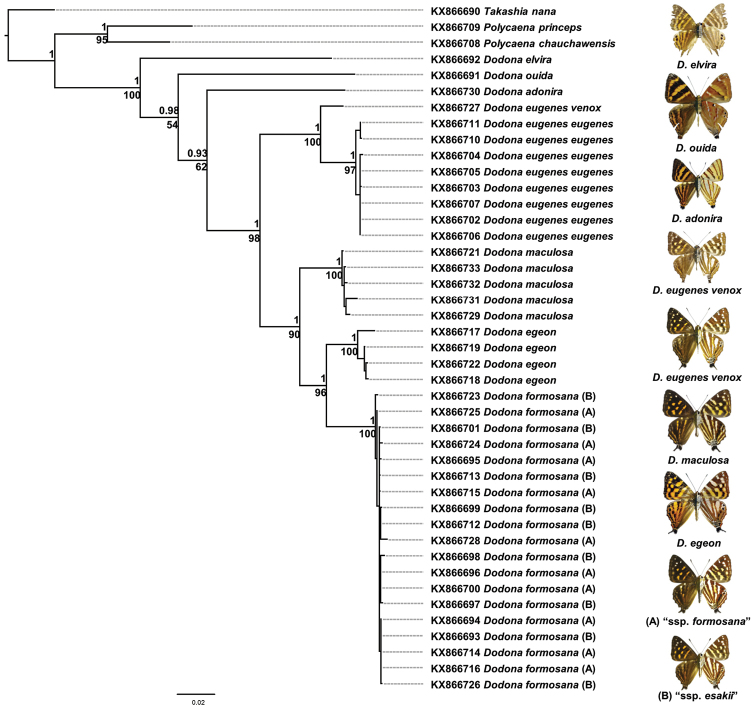
Phylogenetic relationships of sampled *Dodona* butterflies based on BI method. Posterior probabilities are showed above branches and ML bootstrap values are below. **A** ssp. formosana **B** spp. *esakii*.

Comparing male genitalia of ssp. formosana (n = 27) and ssp. esakii (n = 11), no clear difference was recognized (Fig. 1A, B), except a larger size of uncus and valva in ssp. formosana (Fig. 2B, C). However, when the length of phallus, uncus, and valva were measured (ssp. formosana n = 9 and ssp. esakii n = 11), the ratio of uncus/valva and the length of phallus showed no difference between these two groups (t-test, t = -0.9868, d.f. = 18, p = 0.3368; Fig. 2A). Comparing male genitalia of *Dodona* from Taiwan to *Dodona* from other regions (Fig. 1), revealed several distinct differences between the *Dodona* from Taiwan and subspecies of *Dodona eugenes* from other regions: (1) the valva is narrower and longer in the samples from Taiwan (Fig. 1A–D); (2) the costal process is L-shaped in the samples from Taiwan (Fig. 1A, B) versus triangular-shaped from the other regions (Fig. 1C, D); (3) the phallus is upward in all *Dadona* taxa, and the samples from Taiwan show longer and slenderer; (4) the shape of carina penis is gourd-like in all samples, but the specimens from Taiwan represent more broader and longer ones (Fig. 1A, B); (5) the shape of juxta may provide the most useful diagnostic character: the forms of juxta of all examined samples are “X”-shaped (Fig. 1A–D), but the branches are much longer and more slender (Fig. 1A, B) and the terminal end of juxta branch is bifurcated, which is distinct from other subspecies of *D. eugenes* (Fig. 1A–D). Among all the sampled specimens, the *Dodona* samples from Taiwan are most similar in genitalia morphologies to *D. egeon* (Fig. 1E), which possess a wing pattern that is not similar to those found in taxa of *Dodona eugenes* species complex (Fig. 5G, H).

### Phylogeny

A total of 43 specimens was sequenced for COI and COII (Suppl. material 1), and the aligned dataset was 2211 bps in length with only 0.9 % missing data. The phylogenetic relationships inferred by BI and ML methods show concordant species-relationships (Fig. 3; more detail BI and ML topologies were deposited in Suppl. material 3). Most species relationships are strongly supported (BI posterior probability > 0.95 and ML bootstrap value > 85) and only phylogenetic positions of *D. auida* and *D. adonira* are moderately supported (only BI posterior probability > 0.9) (Fig. 3). Interestingly, the taxa currently classified as *Dodona eugenes* did not form a monophyletic group. They are divided into two subunits, with one of them, the *Dodona* samples from Taiwan appeared sister to *D. egeon* with strongly support values (BI = 1.0; ML = 96), whereas ssp. eugenes and ssp. venox were grouped together with strong support (BI = 1.0; ML = 100) (Fig. 3).

### Haplotype network

**Figure 4.**
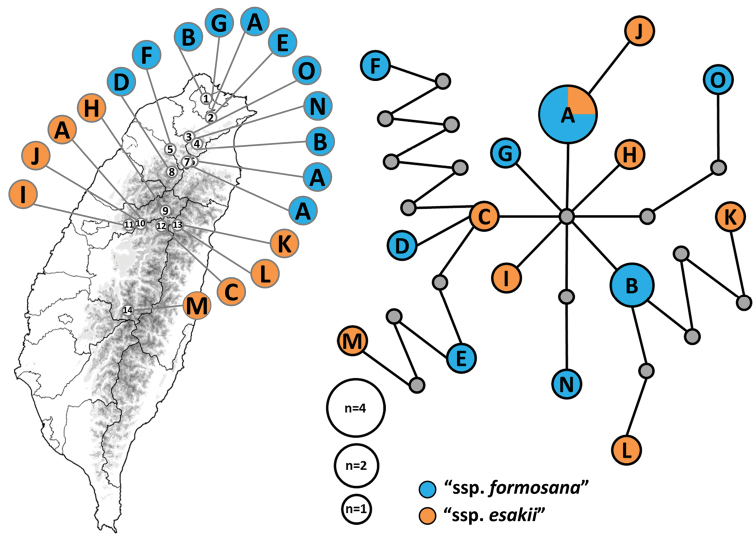
Haplotype network of *Dodona* butterfly in Taiwan. Blue circles represented ssp. formosana, whereas orange circles mentioned ssp. esakii.

Haplotype network was inferred based on an aligned matrix (2211 bps) comprised by 19 *Dodona* individuals from Taiwan. There are 30 variable sites, with 15 haplotypes found in 14 localities. Haplotype A was found in four different localities, including sites in both northern and central Taiwan (Fig. 4; Suppl. material 4). The network structure showed that the populations of ssp. formosana and ssp. esakii are mixed up with no subdivision (Fig. 4).

### Genetic distance

The Kimura-2-Parameter pairwise distance (K2P-distance) between species were calculated and shown on Table 2. The distance among *Dodona* samples ranged from 3.4 % to 9.5 %. The smallest distance was found between *D. egeon* and *D. formosana* (K2P-distance = 0.034), whereas the largest one was between *D. ouida* and *D. elvira* (K2P-distance = 0.095). When comparing *Dodona* to the other examined genera, the K2P-distances are range from 8.2 % to 9.8 %.

**Table 2.** K2P-distances among species of this study.

	1	2	3	4	5	6	7	8	9
1. *Dodona adonira*									
2. *Dodona egeon*	0.073								
3. *Dodona elvira*	0.093	0.079							
4. *Dodona eugenes*	0.079	0.052	0.087						
5. *Dodona formosana*	0.073	0.034	0.085	0.056					
6. *Dodona maculosa*	0.080	0.043	0.089	0.057	0.045				
7. *Dodona ouida*	0.082	0.081	0.095	0.082	0.080	0.088			
8. *Polycaena chauchawensis*	0.093	0.085	0.092	0.088	0.087	0.094	0.086		
9. *Polycaena princeps*	0.095	0.089	0.092	0.097	0.092	0.098	0.098	0.053	
10. *Takashia nana*	0.089	0.084	0.087	0.082	0.084	0.092	0.083	0.061	0.071

## Systematics

Based on the evidence from genitalia morphology and phylogenetic relationships, *Dodona* from Taiwan should be separated from *D. eugenes* and regarded as a distinct species. We therefore raised the *Dodona* butterfly in Taiwan to full species status, *Dodona formosana* Matsumura 1919, stat. rev., The two previously recognized subspecies, spp. *formosana*, and spp. *esakii*, are thus recognized as synonyms (syn. n.).

### Dodona formosana

Taxon classificationAnimaliaORDOFAMILIA

Matsumura, 1919 stat. rev.

Dodona eugenes var. formosana Matsumura, 1919: Thous. Ins. Jap. Vol. 3: 591, pl. 46, f. 4, 5 (Type locality: Shito and Tochosi, Taihoku Pref., North Formosa; Holotype in SEHU).
Dodona eugenes matsumurana Nomura, 1930: Zephrus 2(2): 157–159, pl. 8, f. 1 (Type locality: Oowaki, Chikuto, Shinchiku Pref.; Holotype in SEHU).
Balonca formosana Hirayama, 1939: Genshoku Chrôui Zufu: pl. 22, f. 1, 2 (Type locality: Urai, Taihoku pref., North Formosa).
Dodona eugenes esakii Shirôzu, 1952: Sieboldia 1(1): 23-24, pl. 8, f. 44, 45, 48, 49 (Type locality: Taikokei valley and Heiganzan, Taichû Pref.), syn. n.

#### Material examined.

**Figure 5.**
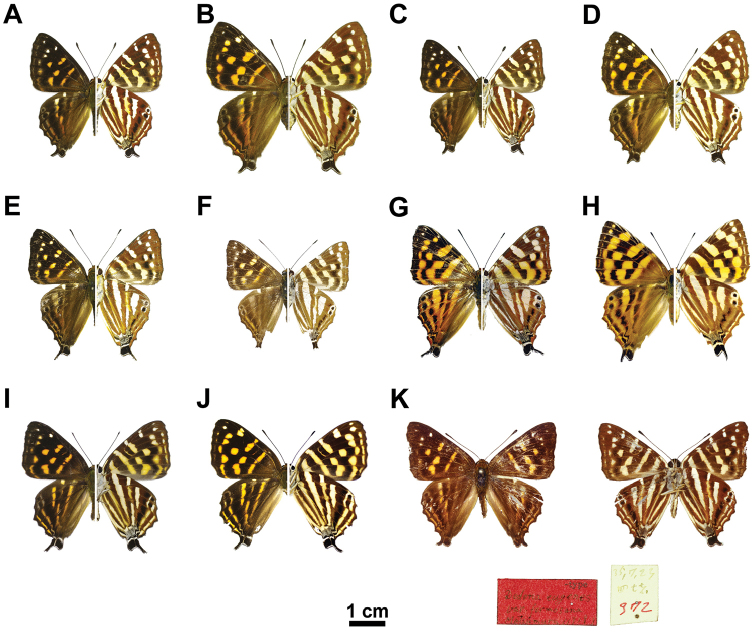
Pinned specimens of *Dodona formosana* and their relatives. **A** *D. formosana* (spp. *formosana*) ♂ **B** *D. formosana* (spp. *formosana*) ♀ **C** *D. formosana* (spp. *esakii*) ♂ **D** *D. formosana* (spp. *esakii*) ♀ **E** *D. eu. eugenes* ♂ **F** *D. eu. venox* ♂ **G** *D. egeon* ♂ **H** *D. egeon* ♀ **I** *D. maculosa* ♂ **J** *D. maculosa* ♀ **K** type of *D. formosana* (SEHU).

***Holotype*** (Fig. 5K). ♂ labelled ‘35.7.23’, ‘Shito [in Chinese]’ (Shito, Pinglin, Xinbei, TAIWAN), ‘972’, ‘type Dodona eugenes var. formosana Matsumura’ (SEHU)

***Additional material.*** 1 ♂ labelled ‘2013. IX.24 (Collect.)’, ‘Mt. Erge Shiding Xinbei’, ‘leg. W. J. Lin, C. W. Huang, C. J. Peng, Y. T. Chen’; 1 ♂ labelled ‘2014. VII.03 (Collect.)’, ‘2014.IX.20 (Eclosion)’, ‘Mt. Erge Shiding Xinbei’, ‘leg. W. J. Lin’, ‘genitalia Dn066’; 1 ♂ labelled ‘2014. V.17 (Collect.)’, ‘2014.VII.09 (Eclosion)’, ‘Daluntou Neihu Taipei’, ‘leg. K. W. Hsiao, Y. M. Hsu’, ‘genitalia Dn034’, ‘14E37’; 1 ♂ labelled ‘2014.VII.07 (Eclosion)’, ‘2014. V.17 (Collect.)’, ‘Daluntou Neihu Taipei’, ‘leg. K. W. Hsiao, Y. M. Hsu’, ‘14E37’; 1 ♂ labelled ‘2014. V.17 (Collect.)’, ‘Daluntou Neihu Taipei’, ‘leg. K. W. Hsiao, Y. M. Hsu’; 1 ♂ labelled ‘2014. V.17 (Collect.)’, ‘2014.VII.09 (Eclosion)’, ‘Daluntou Neihu Taipei’, ‘leg. K. W. Hsiao, Y. M. Hsu’, ‘genitalia lot. Dn038’, ‘14E37’; 1 ♂ labelled ‘2014. V.17 (Collect.)’, ‘2014.VII.09 (Eclosion)’, ‘Daluntou Neihu Taipei’, ‘leg. K. W. Hsiao, Y. M. Hsu’, ‘genitalia lot. Dn039’, ‘14E37’; 1 ♂ labelled ‘2014. V.17 (Collect.)’, ‘Daluntou Neihu Taipei’, ‘leg. K. W. Hsiao, Y. M. Hsu’; 1 ♂ labelled ‘2014. V.17 (Collect.)’, ‘2014.VII.09 (Eclosion)’, ‘Daluntou Neihu Taipei’, ‘leg. K. W. Hsiao, Y. M. Hsu’, ‘genitalia lot. Dn033’, ‘14E37’; 1 ♂ labelled ‘2014. V.24 (Collect.)’, ‘2014.VII.15 (Eclosion)’, ‘Daluntou Neihu Taipei’, ‘leg. W. J. Lin, C. P. Hseuh’, ‘14E50’; 1 ♂ labelled ‘2014. V.24 (Collect.)’, ‘2014.VII.15 (Eclosion)’, ‘Daluntou Neihu Taipei’, ‘leg. W. J. Lin, C. P. Hseuh’, ‘14E50’; 1 ♀ labelled ‘2014.VII.09 (Eclosion)’, ‘2014. V.24 (Collect.)’, ‘Daluntou Neihu Taipei’, ‘leg. W. J. Lin, C. P. Hseuh’, ‘14E50’; 1 ♀ labelled ‘2014. V.24 (Collect.)’, ‘Daluntou Neihu Taipei’, ‘leg. W. J. Lin, C. P. Hseuh’, ‘14E48’; 1 ♀ labelled ‘2014. V.24 (Collect.)’, ‘Daluntou Neihu Taipei’, ‘leg. W. J. Lin, C. P. Hseuh’, ‘14E48’; 1 ♂ labelled ‘2014. V.24 (Collect.)’, ‘Daluntou Neihu Taipei’, ‘leg. W. J. Lin, C. P. Hseuh’; 1 ♂ labelled ‘2014. VIII.03 (Collect.)’, ‘Mt. Erge Shiding Xinbei’, ‘leg. W. J. Lin’, ‘genitalia lot. Dn023’; 1 ♂ labelled ‘2014. VIII.03 (Collect.)’, ‘2014.IX.25 (Eclosion)’, ‘Mt. Erge Shiding Xinbei’, ‘leg. W. J. Lin’, ‘genitalia lot. Dn024’; 1 ♂ labelled ‘2014. VIII.03 (Collect.)’, ‘2014.IX.21 (Eclosion)’, ‘Mt. Erge Shiding Xinbei’, ‘leg. W. J. Lin’, ‘genitalia lot. Dn025’; 1 ♂ labelled ‘2015. VI.27 (Collect.)’, ‘Daluntou Neihu Taipei’, ‘leg. W. J. Lin’, ‘genitalia lot. Dn067’; 1 ♂ labelled ‘2015. VI.27 (Collect.)’, ‘2015.VIII.17 (Eclosion)’, ‘Daluntou Neihu Taipei’, ‘leg. W. J. Lin’, ‘genitalia lot. Dn064’; 1 ♂ labelled ‘2015. VIII.04 (Collect.)’, ‘2015.IX.20 (Eclosion)’, ‘Daluntou Neihu Taipei’, ‘leg. W. J. Lin’, ‘genitalia lot. Dn065’; 1 ♂ labelled ‘2015. VIII.04 (Collect.)’, ‘2015.IX.24 (Eclosion)’, ‘Daluntou Neihu Taipei’, ‘leg. W. J. Lin’, ‘genitalia lot. Dn082’; 1 ♂ labelled ‘2014.VIII.30 (Collect.)’, ‘Mt. Neinaotsui Jianshi Xinchu’, ‘leg. L. H. Wang, J. Y. Liang’, ‘genitalia lot. Dn032’, ‘14H41-1-MA03’; 1 ♂ labelled ‘2014.VIII.30 (Collect.)’, ‘Mt. Neinaotsui Jianshi Xinchu’, ‘leg. L. H. Wang, J. Y. Liang’, ‘genitalia lot. Dn070’, ‘14H41-2-MS01’; 1 ♂ labelled ‘2013.VII.27 (Eclosion)’, ‘2013.VI.20 (Collect.)’, ‘Mt. Malun Hoping Taichung’, ‘leg. W. J. Lin’; 1 ♀ labelled ‘2013.VIII.02 (Eclosion)’, ‘2013.VI.22 (Collect.)’, ‘Mt. Malun Hoping Taichung’, ‘leg. W. J. Lin’; 1 ♂ labelled ‘2013.VIII.10 (Eclosion)’, ‘2013.VI.25 (Collect.)’, ‘Mt. Malun Hoping Taichung’, ‘leg. W. J. Lin’, ‘genitalia lot. Dn027’; 1 ♂ labelled ‘2013.IX.29 (Eclosion)’, ‘Mt. Malun Hoping Taichung’, ‘leg. W. J. Lin’, ‘genitalia lot. Dn010’; 1 ♂ labelled ‘2013. XI.08 (Eclosion)’, ‘2013.XI.29 (Collect.)’, ‘Mt. Malun Hoping Taichung’, ‘leg. W. J. Lin’, ‘genitalia lot. Dn026’; 1 ♂ labelled ‘2014.III.16 (Collect.)’, ‘Mt. Malun Hoping Taichung’, ‘leg. W. J. Lin’, ‘genitalia lot. Dn028’; 1 ♂ labelled ‘2014. IV.01 (Eclosion)’, ‘2014.III.16 (Collect.)’, ‘Mt. Malun Hoping Taichung’, ‘leg. W. J. Lin’, ‘genitalia lot. Dn028’; 1 ♂ labelled ‘2014. X.07 (Eclosion)’, ‘2014.XI.20 (Collect.)’, ‘Mt. Malun Hoping Taichung’, ‘leg. W. J. Lin, C. C. Lin’, ‘genitalia lot. Dn083’; 1 ♂ labelled ‘2014. IV.02 (Collect.)’, ‘Mt. Malun Hoping Taichung’, ‘leg. W. J. Lin, C. J. Chang, Y. H. Lin, M. F. Chou’, ‘genitalia lot. Dn059’; 1 ♂ labelled ‘2014. IV.02 (Collect.)’, ‘Mt. Malun Hoping Taichung’, ‘leg. W. J. Lin, C. J. Chang, Y. H. Lin, M. F. Chou’, ‘genitalia lot. Dn084’; 1 ♂ labelled ‘2014.IX.28 (Collect.)’, ‘2014. VIII.25 (Eclosion)’, ‘Songmao Forest Road Hoping’, ‘leg. W. J. Lin, C. W. Huang, Y. M. Hsu, Y. H. Lin’, ‘genitalia lot. Dn085’; 1 ♂ labelled ‘2014. XI.11 (Eclosion)’, ‘2014.X.07 (Collect.)’, ‘Mt. Malun Hoping Taichung’, ‘leg. W. J. Lin, C. C. Lin’, ‘genitalia lot. Dn068’; 1 ♂ labelled ‘2015.VII.05 (Collect.)’, ‘Taiwan No. 8 Highway 119.5K Xiulin Hualien’, ‘leg. L. Huang’, ‘genitalia lot. Dn062’, ‘DNA lot. Rd030’.

#### Redescription of adults.

***Male*** (Fig. 1A, B; 5A, C; Suppl. material 5). *Head*: Frons hairy, dark brown with a white band, edge with white laterally and ventrally. Chaetosemata forming a pair of transverse patches behind antennae. Eye semi-oval, sparsely hairy. Labial plapus porrect, white with distal tip dark brown, third segment extremely short. *Thorax*: Brown dorsally, white ventrally. Legs basically white, with tibia and tarsus clothed with yellow and brown scales; foretarsus with all tarsomeres fused. Length of forewing 2.871-3.569 mm (3.265 ± 0.172mm; n = 49). *Forewing*: Ground color of upper side brown. Fringe brown, but white in cell CuA_2_ and 1A+2A. Central symmetry system with proximal band forming an orange band; distal band discrete, displaced in cell M_3_ and CuA_1_. Apical spots white, submarginal spots orange, reduced occasionally. Ground color of underside brick red. Central symmetry system with proximal band and distal band similar to upper side but spots white to light yellow, discal spot slim. Submarginal spots white to light yellow, forming a discrete line, displaced in cell M_1_, M_2_, and M_3_. Parafocal element white, displaced and occasionally reduced in cell M_1_ and the following cell. *Hindwing*: Ground color of upper side brown, besides discal band, elements of central symmetry system bent inwards in cell CuA_2_. Fringe white checkered with brown. Central symmetry system with proximal band forming an orange band, occasionally faint; distal band orange, displaced in cell M_1_, M_2_ and M_3_, discal band slim, reduced occasionally. Submarginal spots white in cell M_1_ and M_2_, orange and displaced in cell M_3_ and the following cells. Parafocal element nearly parallel to submarginal spots, extended to tornus. Tail-like projection of cell Cu_2_ black, with fringe white forward but white mixed with brown behind. Lobe-like projection of cell 1A+2A black, with fringe brown mixed with white. Ground color of underside brick red, arrangement of central symmetry system similar to upper side but elements in cell 1A+2A and 3A more visible. Central symmetry system with proximal band slightly silver; distal band slightly silver, displaced in cell M_1_, M_2_ and M_3_, discal band light yellow, faint occasionally. Submarginal spots and paraforcal band white, border ocelli black in cell M_1_ and M_2_, black mixed with white in cell CuA_2_ and 1A+2A, elements in cell M_1_ and M_2_ forming two “eyespots”. *Genitalia*: With 9^th^ and 10^th^ segments fused, forming a complete ring; tegumen triangular in lateral view; vinculum evenly narrow; saccus reduced; uncus hook-like and down-curved posteriorly; branchia slender, swollen basally, bent medially; valva broader dorsally, a projection present at distal end of dorsal margin, sparse setae along margin; phallobase gradually tapering caudally; phallus up-curved; carina penis elongate and wavy laterally; cornuti present in the form of sclerotized band bearing minute spicules; juxta “X”-shaped, anterior branch broader, dorsal posterior branch evenly wide, ventral posterior branch with a bifurcate tip.

***Female*** (Fig. 5B, D; Suppl. material 5). *Head*: Structure and color pattern similar to that of male. ***Thorax***: Structure and color pattern similar to that of male, but foretarsus without tarsomeres fused. Length of forewing 2.994–3.843 mm (3.404 ± 0.178 mm; n = 43). *Wing*: Configuration similar to that of male, but ground color slightly brighter than that of male. Markings much more prominent than those of male. Termen more rounded than that of male. *Genitalia*: Papillae anales sparsely setose, sclerotized, forming a pair of rounded triangles. Posterior end of ductus bursae forming a sclerotized tube. Ductus bursae membranous anteriorly, with ductus seminalis joining dorsally and immediately cephalic to sclerotized tube. Corpus bursae ovoid, signa double, forming small invaginated, oval projections.

#### Diagnosis.

Wing pattern of *Dodona formosana* is similar to *D. eugenes* and *D. maculosa* Leech 1890, in sharing small markings and narrow stripes (Fig. 5), but can be distinguished by following characters: (1) The silver stripes on the underside of the hindwing is more prominent in *D. eugenes* (Fig. 5E, F); the spots on the wing upper side is orange in *D. formosana* (Fig. 5A–D), but light orange or yellow is presented in *D. eugenes* (Fig. 5E, F). (2) The yellow spots posterior to cell M on the underside of the forewing are brighter in *D. formosana* (Fig. 5A–D), the proximal band on the upper side of the hindwing is less prominent in *D. formosana* (Fig. 5A–D).

Male genitalia of *D. formosana* is quite different from that of *D. eugenes* and *D. maculosa*, especially in the following characters (Fig. 1A–D, F): (1) the valva is narrower and longer in *D. formosana*, its long axis is nearly two times as long as short axis; (2) the costal process is L-shaped in *D. formosana*, whereas it is triangular in *D. eugenes* and *D. maculosa*; (3) the juxta of *D. formosana* and *D. eugenes* are nearly divided in the middle (Fig. 1A–D), whereas they are strongly fused in *D. maculosa* (Fig. 1F); besides, the branches of juxta are slenderer in *D. formosana* (Figs 1A–B) than *D. eugenes* (Fig. 1C–D); (4) the phallus is longer and slenderer in *D. formosana*; (5) the tip of carina penis is longer and down-curved in *D. maculosa* (Fig. 1F), and bifurcated in *D. eugenes* (Fig. 1C–D).

#### Bionomics.

Eggs are laid singly, or a few in a small cluster on the leaf or branch, also in debris near hostplant. Larval hostplants are *M. seguinii* and *M. africana*. The 1^st^ and 2^nd^ instar larvae devour young leaves or scratch the mesophyll of old leaves. Final instar larva pupates on the underside of leaf (Suppl. material 6).

#### Distribution.

This species is endemic in Taiwan.

## Discussion

Based on morphology of male genitalia (Fig. 1) and phylogenetic relationships (Fig. 3), it can be concluded that *D. formosana* represents a distinct species and not a subspecies of *Dodona eugenes*. The most closely related species of *D. formosana* is *D. egeon*, whereas *D. eugenes* is the sister to a clade including *D. maculosa*, *D. egeon*, and *D. formosana*. Two subspecies of *D. eugenes*, spp. *venox* and spp. *eugenes*, are considered conspecific as the morphologies of their male genitalia are similar to each other (Fig. 1C, D), and they represent a monophyletic assemblage (Fig. 3). It is worth noticing that the K2P-distance between spp. *venox* and spp. *eugenes* is quite high (K2P-distance = 0.026), near the species-level distance proposed by Hebert et al. (2004). Thus further work is needed to clarify species status of *D. eugenes*, which as currently understood is widespread from India to Southeast Asia, with populations fragmented on mountains and islands.

The characters of the male genitalia (Fig. 1; Fig. 2) and the inferred haplotype network are both showed that no further division can be made between northern and central populations of *D. formosana* (Fig. 4). This suggests there is no clear geological boundary between previously recognized subspecies. The generally larger body size of northern populations of *D. formosana* may be caused by other factors, such as different utilization of local hostplants. Our preliminary test have shown that the northern populations of *D. formosana* have different number of larval instars when they fed on *M. sequinii* versus *M. africana* (Lin 2016). This suggests that different food plants may alter larval physiology (Bocaz et al. 2003).

## Conclusions

The *Dodona* butterfly in Taiwan should be regarded as an endemic species, *Dodona formosana*, distinct from *D. eugenes*. The present work and a previous study (Callaghan 2009) both point out that *Dodona eugenes*, widely distributed in East Asia, is not a monophyletic species. Combining evidence from genitalia characters and molecular sequences provides effective clarification on solving species-level problem for species complex containing superficially similar species.

## Supplementary Material

XML Treatment for Dodona formosana

## Supplementary materials

Supplementary material 1 Sampling information Data type: species data Explanation note: Sample list and sampling information of this study. This is an open access article distributed under the terms of the Creative Commons Attribution License (CC BY 4.0), which permits unrestricted use, distribution, and reproduction in any medium, provided the original author and source are credited.Li-Wei Wu, Wen-Jie Lin, Yu-Feng Hsu

Supplementary material 2 Primer information Data type: molecular data Explanation note: Primers used in this study. This is an open access article distributed under the terms of the Creative Commons Attribution License (CC BY 4.0), which permits unrestricted use, distribution, and reproduction in any medium, provided the original author and source are credited.Li-Wei Wu, Wen-Jie Lin, Yu-Feng Hsu

Supplementary material 3 Phylogenetic trees Data type: molecular data Explanation note: Topologies inferred by BI and ML methods based on COI and COII. S3a: BI phylogeny; S3b: ML phylogeny. This is an open access article distributed under the terms of the Creative Commons Attribution License (CC BY 4.0), which permits unrestricted use, distribution, and reproduction in any medium, provided the original author and source are credited.Li-Wei Wu, Wen-Jie Lin, Yu-Feng Hsu

Supplementary material 4 Haplotype information Data type: occurence Explanation note: Variable sites of 15 *Dodona* haplotypes among 14 sampled localities in Taiwan. This is an open access article distributed under the terms of the Creative Commons Attribution License (CC BY 4.0), which permits unrestricted use, distribution, and reproduction in any medium, provided the original author and source are credited.Li-Wei Wu, Wen-Jie Lin, Yu-Feng Hsu

Supplementary material 5 Morphological genitalia Data type: species data Explanation note: Additional male and female genitalia of *Dodona formosana*. **S5a**: **A** Dn032 **B** Dn039 **C** Dn064 **D** Dn067 **E** Dn082 **F** Dn026 **G** Dn059 **H** Dn083 **I** Dn090; **S5b**: Dn055. This is an open access article distributed under the terms of the Creative Commons Attribution License (CC BY 4.0), which permits unrestricted use, distribution, and reproduction in any medium, provided the original author and source are credited.Li-Wei Wu, Wen-Jie Lin, Yu-Feng Hsu

Supplementary material 6 Images Data type: species data Explanation note: Larval stages of *Dodona formosana*. **A** eggs **B** first instar **C** second instar **D** third instar **E** fourth instar **F** pupa. This is an open access article distributed under the terms of the Creative Commons Attribution License (CC BY 4.0), which permits unrestricted use, distribution, and reproduction in any medium, provided the original author and source are credited.Li-Wei Wu, Wen-Jie Lin, Yu-Feng Hsu
