# Supplementary material for: A distinct species, Dodona formosana, detected in the Dodona eugenes species complex: clarification of the taxonomic status of the Punch butterfly in Taiwan
Source: Zookeys. 2018 Feb 8;(736):59–77. doi: 10.3897/zookeys.736.22062 (PMC5904550; doi:10.3897/zookeys.736.22062)
Supplement: Supplementary material 3 — Phylogenetic trees [file zookeys-736-059-s003.pdf]

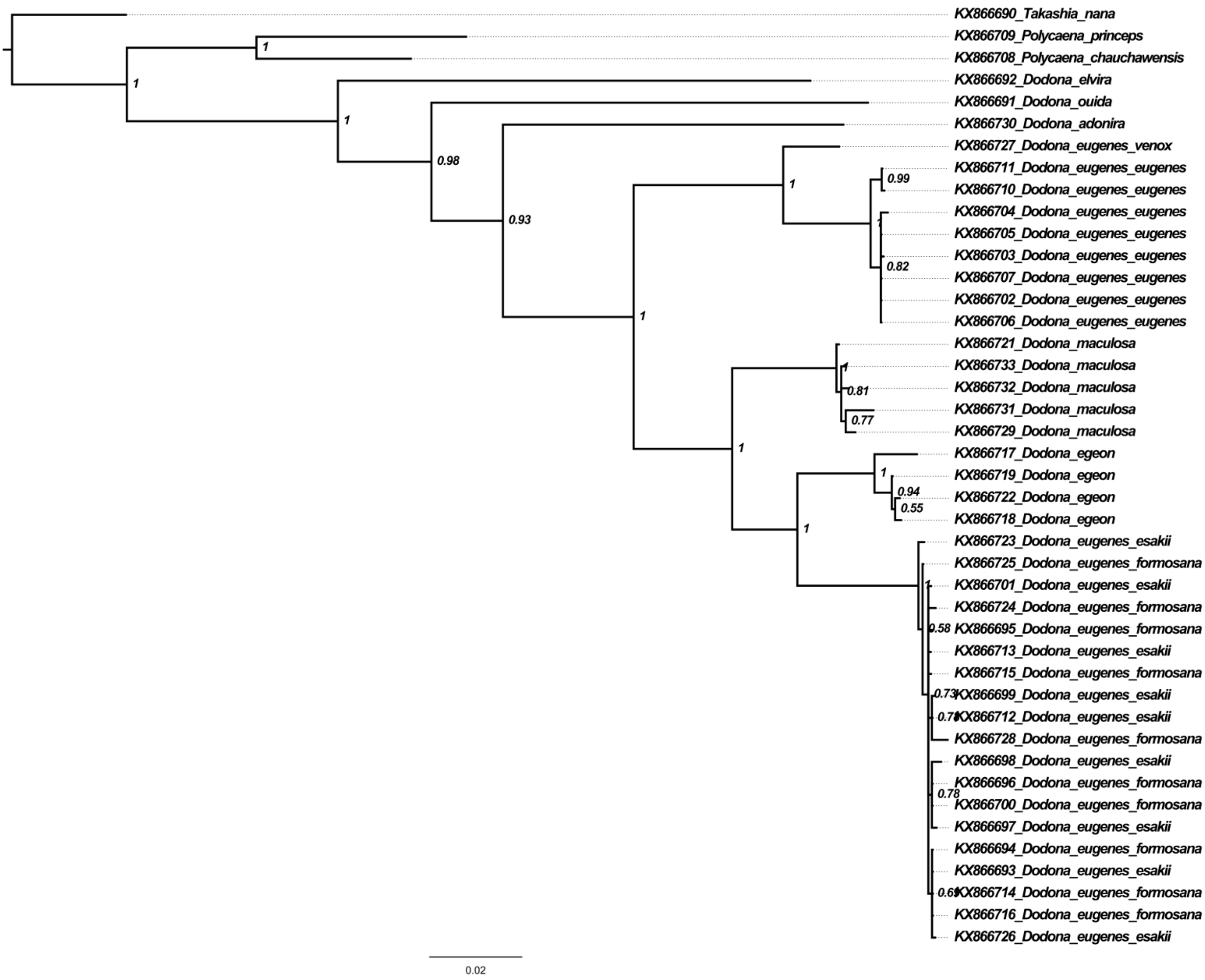

Supplementary file 3a. Bayesian tree inferred based on mitochondrial *cox1* and *cox2* genes.

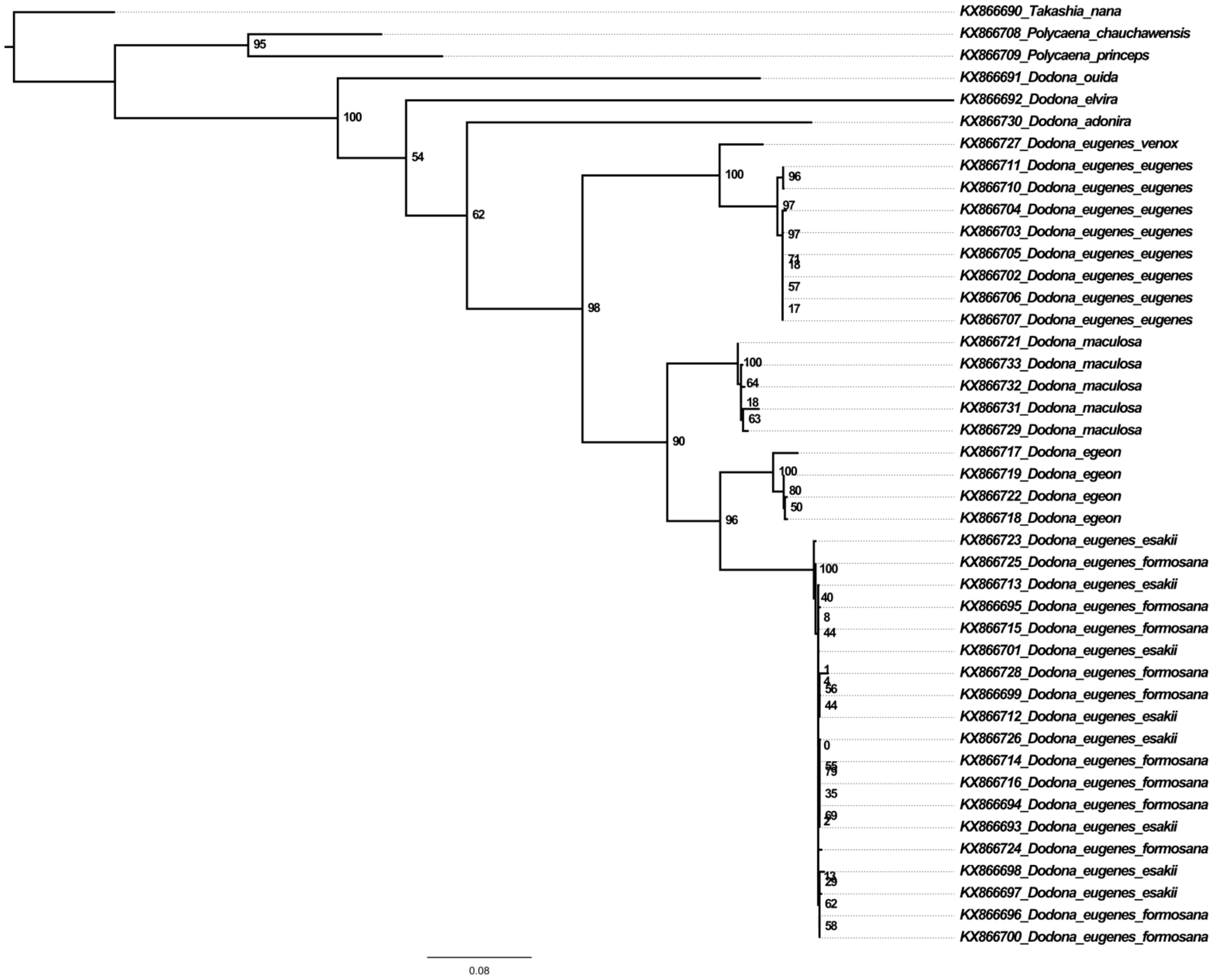

Supplementary file 3b. ML tree inferred based on mitochondrial *cox1* and *cox2* genes.
