# Supplementary material for: A distinct species, Dodona formosana, detected in the Dodona eugenes species complex: clarification of the taxonomic status of the Punch butterfly in Taiwan
Source: Zookeys. 2018 Feb 8;(736):59–77. doi: 10.3897/zookeys.736.22062 (PMC5904550; doi:10.3897/zookeys.736.22062)

**
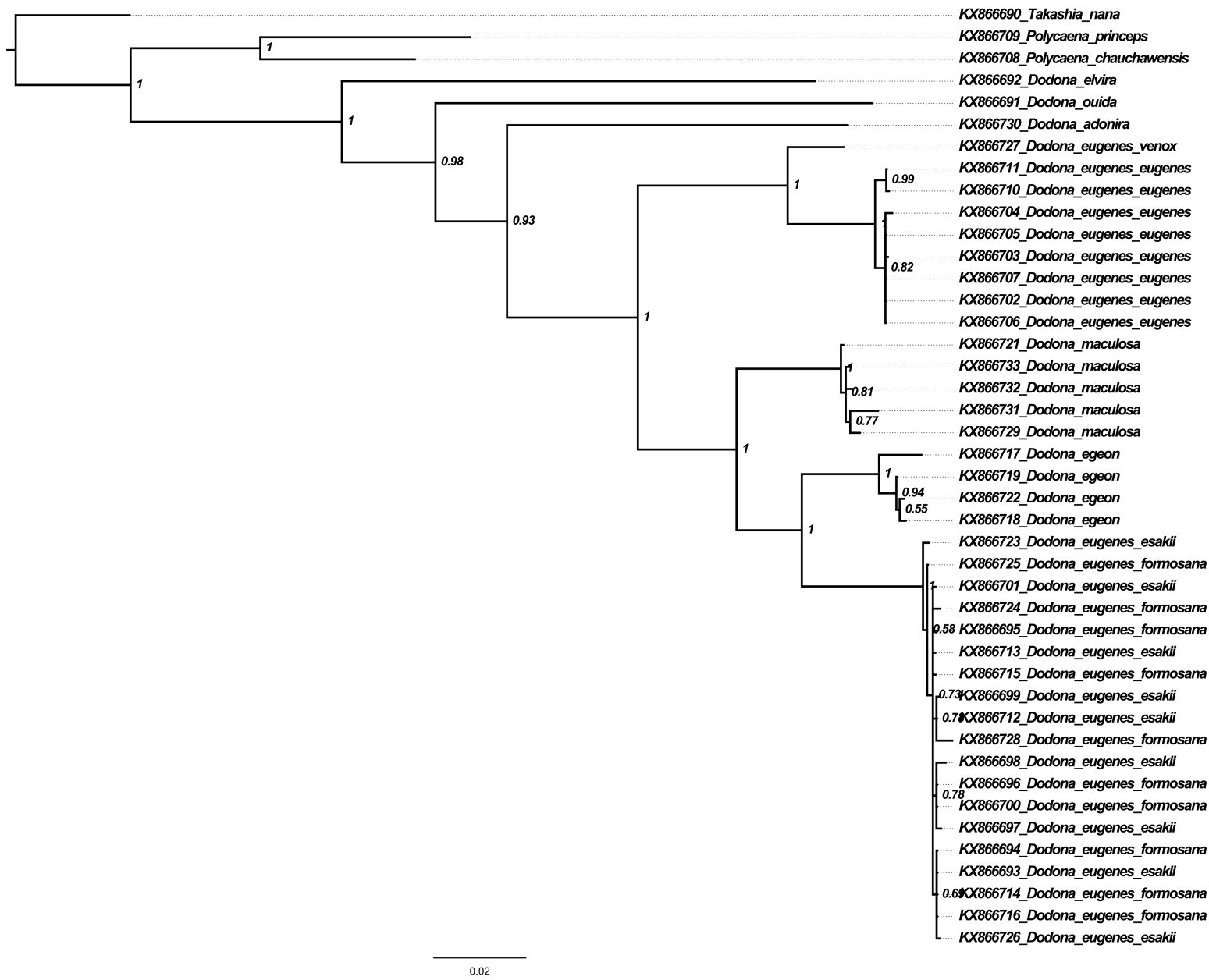
Figure S1.** Bayesian inference tree constructed by mitochondria DNA COI and COII.

**Figure S2.** Maximum likelihood tree constructed by mitochondria DNA COI and COII.
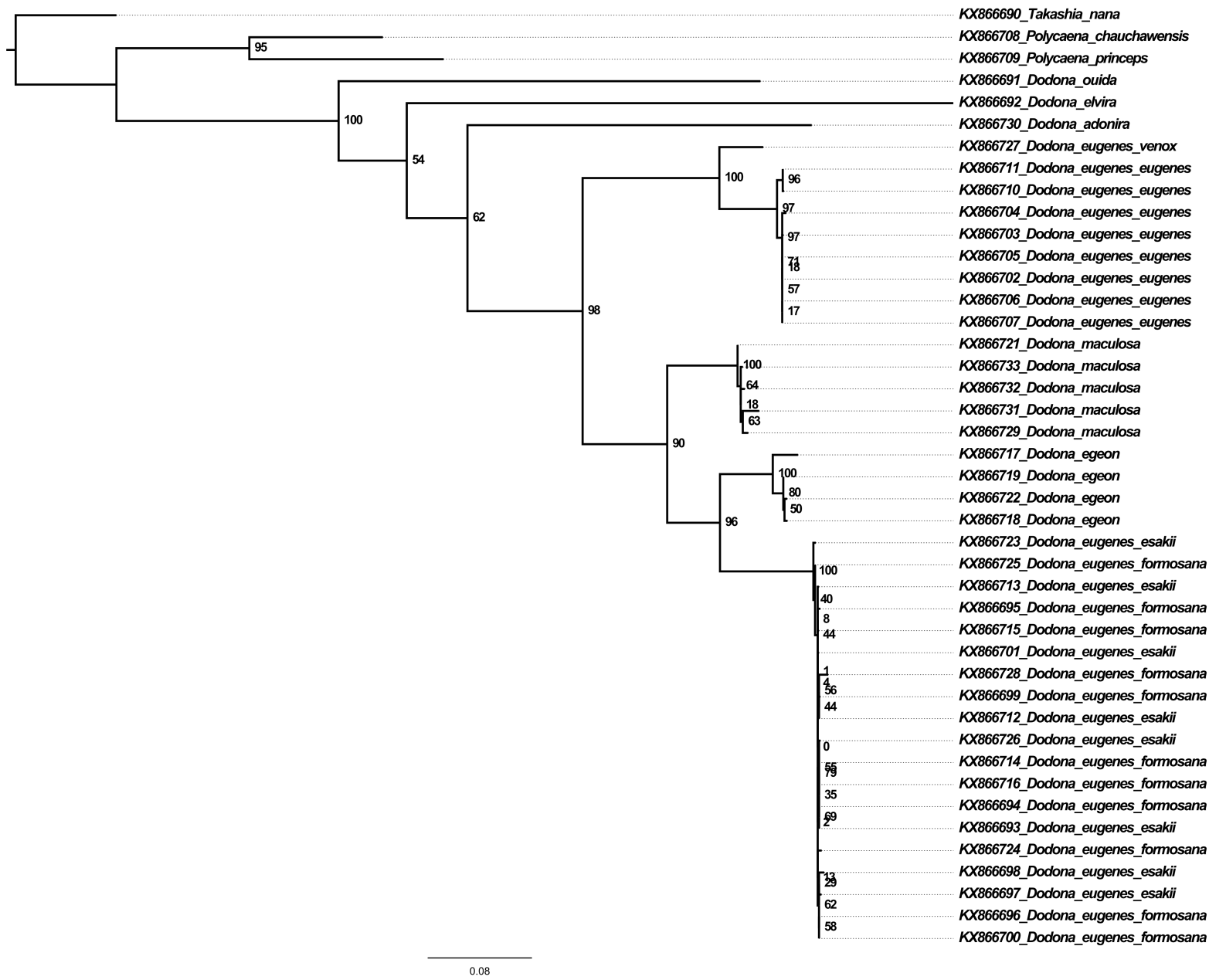

Supplement: Supplementary material 4 — Haplotype information [file zookeys-736-059-s004.docx]
