# Supplementary material for: A distinct species, Dodona formosana, detected in the Dodona eugenes species complex: clarification of the taxonomic status of the Punch butterfly in Taiwan
Source: Zookeys. 2018 Feb 8;(736):59–77. doi: 10.3897/zookeys.736.22062 (PMC5904550; doi:10.3897/zookeys.736.22062)
Supplement: Supplementary material 5 — Morphological genitalia [file zookeys-736-059-s005.pdf]

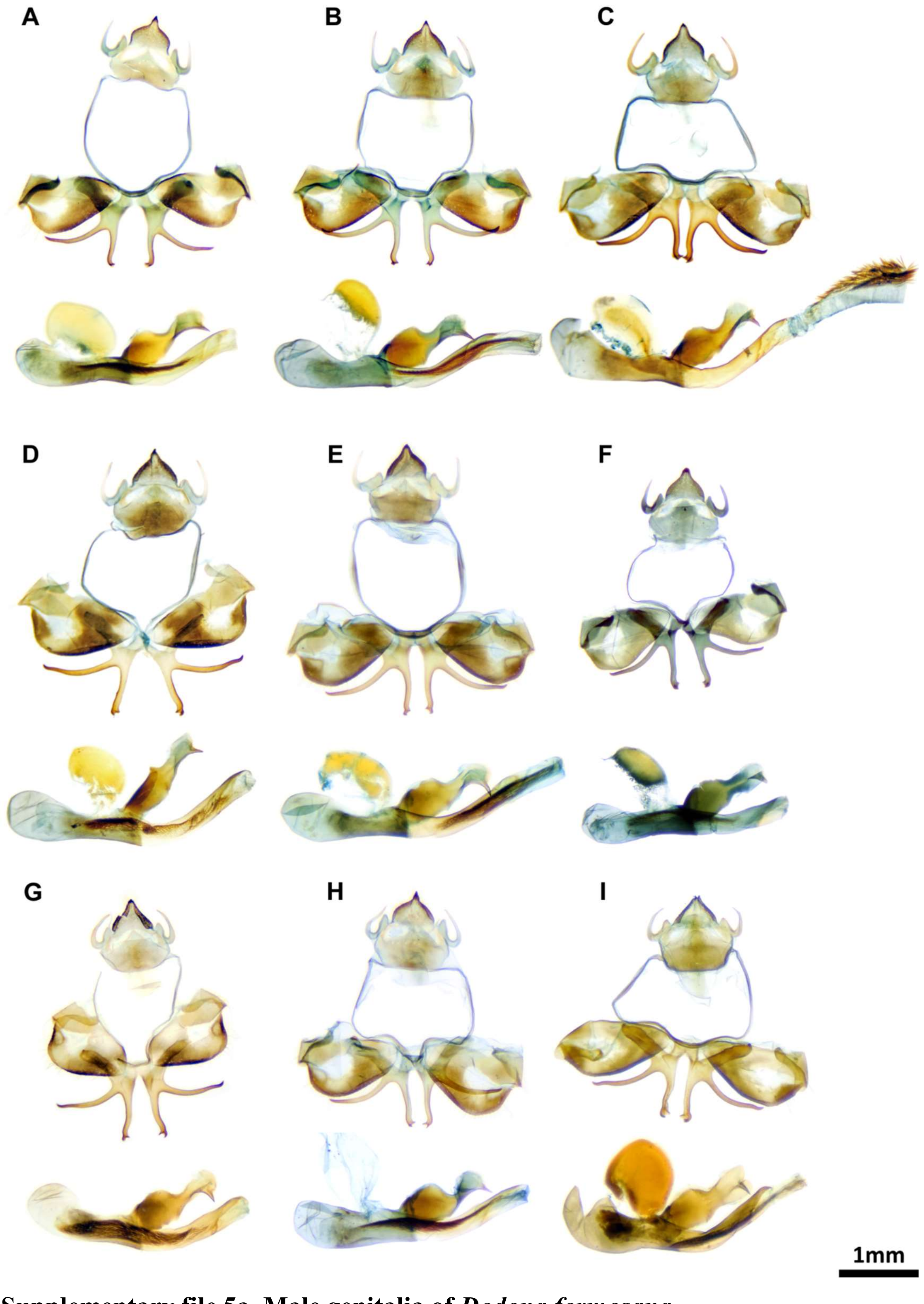

Supplementary file 5a. Male genitalia of *Dodona formosana*.
A “ssp. *formosana*” (Dn032) **B** “ssp. *formosana*” (Dn039)
C “ssp. f*ormosana*” (Dn064) **D** “ssp. *formosana*” (Dn067)
E “ssp. *formosana*” (Dn082) **F** “ssp. *esakii*” (Dn026) **G** “ssp. *esakii*” (Dn059)
H “ssp. *esakii*” (Dn083) **I**, “ssp. *esakii*” (Dn090).

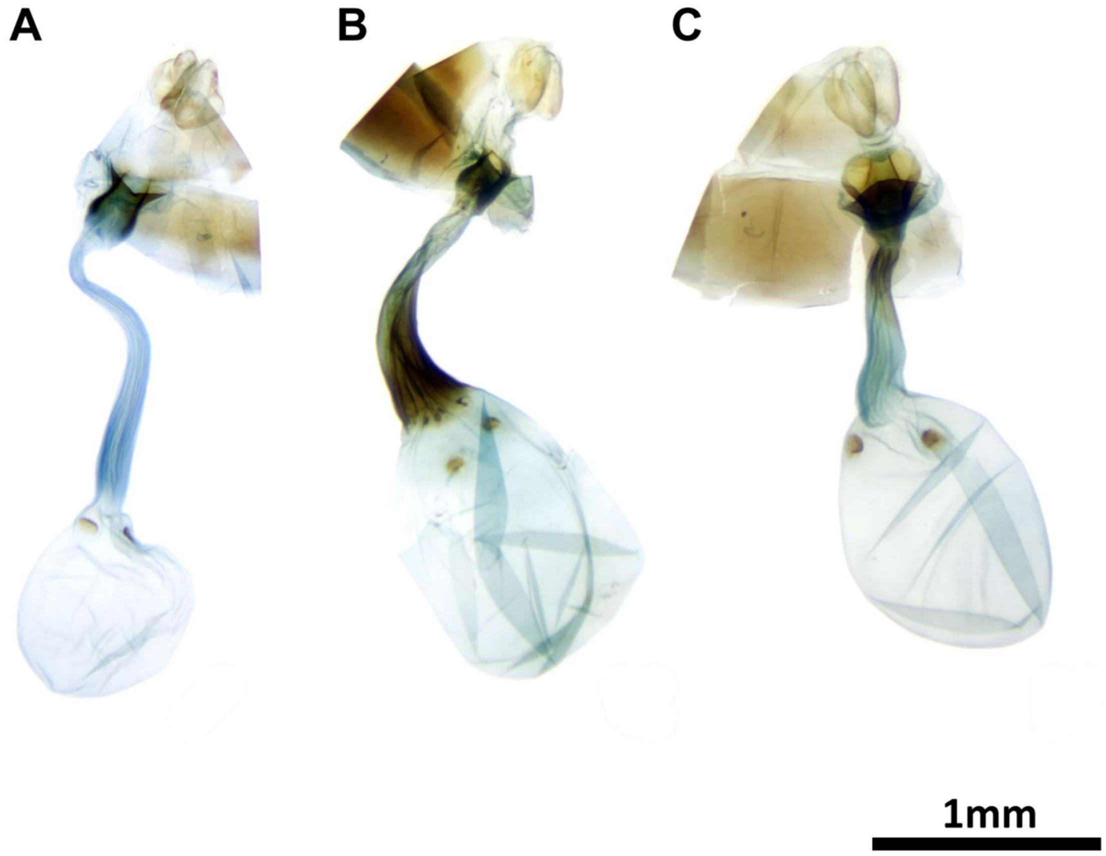

Supplementary file 5b. Female genitalia of genus *Dodona*.
A *D. formosana* (Dn055) **B** *D. egeon* (Dn069) **C** *D. maculosa* (Dn020).
