# Supplementary material for: A distinct species, Dodona formosana, detected in the Dodona eugenes species complex: clarification of the taxonomic status of the Punch butterfly in Taiwan
Source: Zookeys. 2018 Feb 8;(736):59–77. doi: 10.3897/zookeys.736.22062 (PMC5904550; doi:10.3897/zookeys.736.22062)
Supplement: Supplementary material 6 — Images [file zookeys-736-059-s006.pdf]

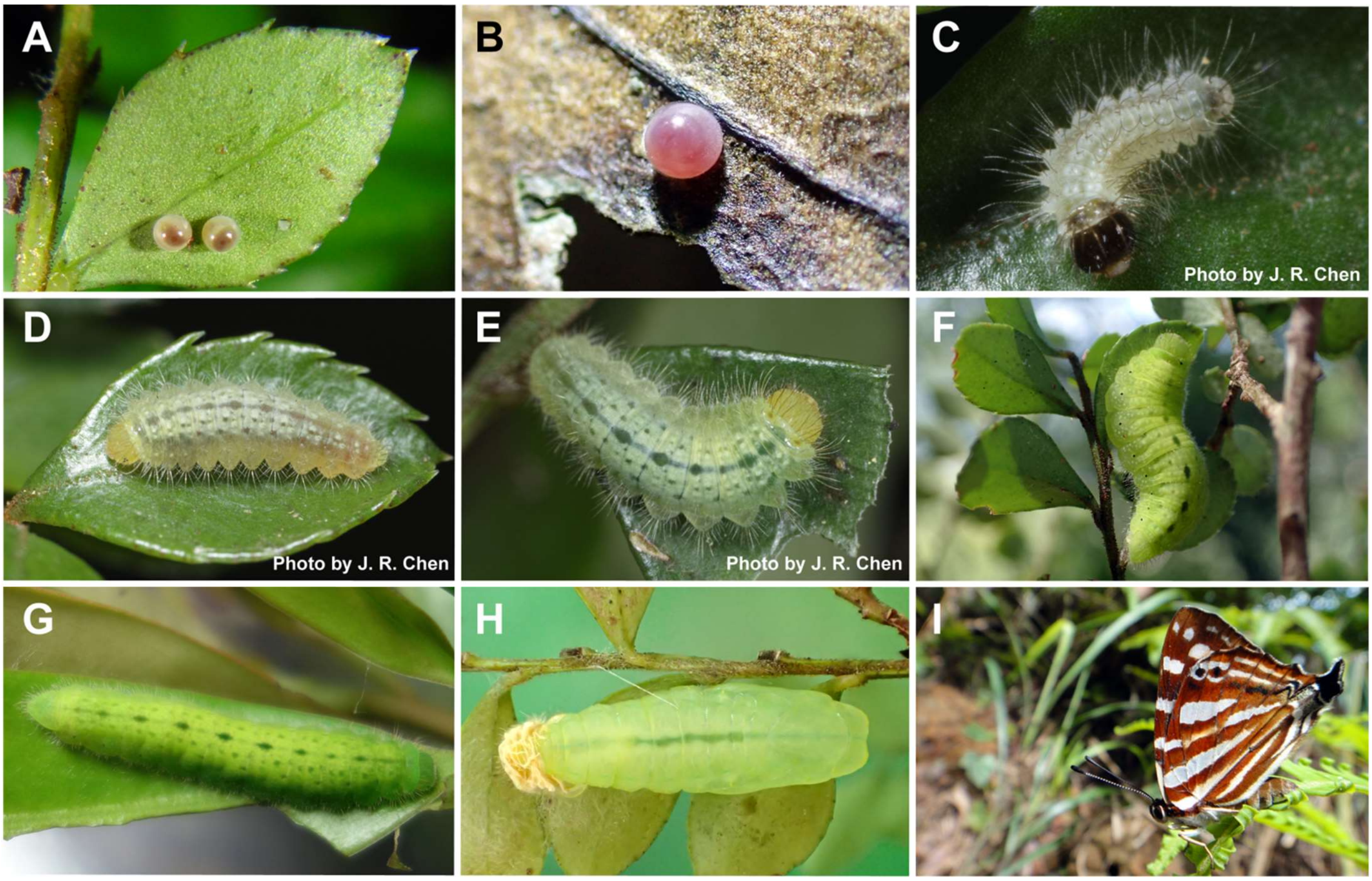

Supplementary file 6. Life history of *Dodona formosana*.

A eggs (laid on hostplant) **B** eggs (laid on materials closed to hostplant) **C** first instar larva **D** second instar larva **E** third instar larva **F** fourth instar larva **G** fifth instar larva **H** pupa **I** adult.
